# The Contribution of Microglia to Neuroinflammation in Parkinson’s Disease

**DOI:** 10.3390/ijms22094676

**Published:** 2021-04-28

**Authors:** Katja Badanjak, Sonja Fixemer, Semra Smajić, Alexander Skupin, Anne Grünewald

**Affiliations:** 1 Luxembourg Centre for Systems Biomedicine, University of Luxembourg, L-4367 Esch-sur-Alzette, Luxembourg; katja.badanjak@uni.lu (K.B.); sonja.fixemer@uni.lu (S.F.); semra.smajic@uni.lu (S.S.); alexander.skupin@uni.lu (A.S.); 2 Luxembourg Centre for Neuropathology (LCNP), L-3555 Dudelange, Luxembourg; 3 Department of Neuroscience, University California San Diego, La Jolla, CA 92093, USA; 4 Institute of Neurogenetics, University of Lübeck, 23562 Lübeck, Germany

**Keywords:** neuroinflammation, microglia, brain, neurodegeneration, animal models, iPSC, Parkinson’s disease

## Abstract

With the world’s population ageing, the incidence of Parkinson’s disease (PD) is on the rise. In recent years, inflammatory processes have emerged as prominent contributors to the pathology of PD. There is great evidence that microglia have a significant neuroprotective role, and that impaired and over activated microglial phenotypes are present in brains of PD patients. Thereby, PD progression is potentially driven by a vicious cycle between dying neurons and microglia through the instigation of oxidative stress, mitophagy and autophagy dysfunctions, a-synuclein accumulation, and pro-inflammatory cytokine release. Hence, investigating the involvement of microglia is of great importance for future research and treatment of PD. The purpose of this review is to highlight recent findings concerning the microglia-neuronal interplay in PD with a focus on human postmortem immunohistochemistry and single-cell studies, their relation to animal and iPSC-derived models, newly emerging technologies, and the resulting potential of new anti-inflammatory therapies for PD.

## 1. Introduction

### 1.1. Parkinson’s Disease

Parkinson’s disease (PD) is one of the most common neurodegenerative disorders with prevalence of around 1% in people aged above 65 years [1]. It is caused by a chronic and progressive loss of dopaminergic neurons in the substantia nigra (SN) pars compacta (pc) [2]. Despite great advances in research, it is still an elusive field of medical science, with around 10% of all cases being of genetic and others of idiopathic origin [3]. Genetic forms of PD include mutations in 23 loci, some of which have been studied intensely in the past two decades: *LRRK2* (*PARK8*), *SNCA* (*PARK1*), *PRKN* (*PARK2*), *PINK-1* (*PARK6*), and *DJ-1* (*PARK7*) [4,5]. Age is considered a major risk factor for the development of PD [6,7]. However, in addition, the genetic predisposition and exposure to environmental factors contribute to pathogenesis, making PD an age-related multifactorial disease. Environmental factors such as herbicides or pesticides can induce oxidative stress, DNA damage, and neuronal cell death [8]. The demise of dopaminergic neurons leads to lower levels of dopamine that is released into the striatum and thus, with progression of the disease, patients endure characteristic motor symptoms (tremor, rigidity, bradykinesia), cognitive decline (dementia) and even psychiatric signs (depression, apathy, anxiety) coupled with constipation and sleep disturbances [1,9,10].

A first clue to what might be the underlying mechanism of parkinsonism symptoms came unexpectedly in 1982 from a group of drug addicts who, after injection of a synthetic heroin, began experiencing symptoms commonly seen in PD patients. The drug was found to be 1-methyl-4-phenyl-1,2,3,6-tetrahydropyridine (MPTP). MPTP, when transformed into its ion 1-methyl-4-phenylpyridinium (MPP+), acts as a highly potent initiator of neuronal cell death through inhibition of complex 1 of the respiratory chain machinery in mitochondria [11,12]. Besides the demise of dopaminergic neurons and mitochondrial dysfunction, other molecular pathologies have been linked to PD since the late 20th century. They include aggregation of α-synuclein, formation of α-synuclein-containing Lewy bodies and neurites, somatic mtDNA alterations, autophagy dysfunction, and activation of microglia cells [13,14,15,16,17].

As of today, most common therapies are purely symptomatic, and they usually include substitution of dopamine by administering the drug Levodopa. Current treatments also have their drawbacks: 1. they only relieve some of the symptoms; 2. they do not slow the progression of the disease; 3. they have limited long-term efficacy. Therefore, PD researchers have turned their focus on how to diagnose the disease during its early phases. Motor symptoms occur at a rather late stage of the disease—only after 50% of dopaminergic neurons have been lost, patients experience impaired movement [18]. Thus, there is hope that with the discovery of early-stage biomarkers, we will be able to target molecular impairments and inflammatory phenotypes during the prodromal phase of the disease, thereby preventing the worst motor symptoms [19]. Among others, promising biomarkers would be indicators of inflammation, cytokines and chemokines, released by microglia into the extracellular space.

### 1.2. Microglia

Microglia are immune cells of the brain, representing the neural tissue’s defense system [20]. Microglia cells were first discovered more than 100 years ago by Pío Del Río Hortega, using a silver carbonate staining method which allowed identifying them in brain tissue samples. He called microglia “the true third element” in addition to neurons and astrocytes [21]. He described microglia as a heterogeneous cell type with various morphologies ranging from highly ramified to ameboid shaped cells providing a first indication for the existence of microglia sub-populations [22].

During fetal development, microglia migrate from the yolk sac as primitive macrophages to the central nervous system’s (CNS) neuroepithelium. In the CNS, they constitute up to 12% of all cells, and their density changes depending on the brain region [23]. Early studies in mice showed that bone-marrow-derived hematopoietic cells move to the CNS, where they differentiate into microglia-like cells. Accordingly, microglia cannot be replenished from the internal CNS pool but peripheral cells need to be recruited to maintain a sufficient number of microglia [24]. By contrast, these studies employed whole-body irradiation techniques, which led to impaired blood-brain barrier (BBB) integrity, giving rise to peripheral cell entry. With innovative conditional cell depletion techniques, it was recently shown that microglia have the ability to self-renew, and that interleukin-1 (IL-1) signaling is enabling this process [25]. Just like the abundance of microglia is region-specific, microglial morphology varies from brain area to brain area. In a resting state, microglia survey the brain microenvironment and show ramified morphology. Surveillance encompasses multiple functions: clearance of accumulated or deteriorated neuronal and tissue elements, dynamic interaction with neurons whilst regulating the synaptic pruning process, and maintaining overall brain homeostasis [26,27]. Once activated upon brain damage and certain host or non-host stimuli, microglia are quickly undergoing a morphology change into an ameboid-like form, coupled with the release of inflammatory molecules, cytokines and chemokines. With regard to their activation, microglia are commonly divided into two classes: M1 (pro-inflammatory) or M2 (anti-inflammatory). Even though, by now, it is known that the states of activation are much more heterogeneous and diverse. 

New approaches are being developed to determine sub-populations of microglia, mostly through single-cell gene expression studies and by determining fine morphological differences using computational methods [28,29,30]. With age, microglia tend to express more IL-1β and they become more phagocytic in nature compared to microglia from younger brains [31,32,33]. These phenotypic changes over time can influence their ability to function normally and attain the neuronal homeostasis and support. Eventually, an accumulation of non-functional, senescent microglia could contribute to irreversible and progressive neurodegeneration in PD.

## 2. Human Post-Mortem Tissue Studies

The first neuropathological evidence suggesting an involvement of microglia in PD was published in 1988 [14]. Compared to age-matched non-neurologic cases, the authors observed an enrichment of reactive microglia in post-mortem SN samples from PD patients characterized by the activation of human major histocompatibility class II (MHC2) proteins which function in antigen presentation and inflammatory signalling. While these MHC2-positive cells covered the whole range of microglia morphologies, the reactive ameboid shape was strongly enriched indicating the association with a neuropathological activity. 

A more recent study confirmed these observations in post-mortem SN tissue of PD patients and additionally described MHC2-positive microglia in the putamen, trans-entorhinal cortex, cingulate cortex and the temporal cortex of these individuals [34]. Further analysis in PD post-mortem brain tissue also revealed a correlation between ameboid shaped microglia appearance and α-synuclein pathology in the SN and the hippocampus (HPC), as well as a SN-specific Toll-like receptor 2 upregulation in these MHC2-positive microglia. Altogether, this work implicated a region-dependent and possibly disease stage-dependent microglia heterogeneity in PD [34].

To complement these morphology-based analyses of microglia heterogeneity on the transcriptional level, single-cell laser capture microscopy was applied to microglia in post-mortem PD brains [35]. This approach allowed exclusively collecting microglia, thereby creating an advantage over common bulk RNA analyses of brain homogenates, which are either contaminated by other cell types or exhibit sorting-induced skewed RNA profiles. The single-cell study revealed that the transcriptional profiles of microglia differ between brain regions (SN, HPC) in both control subjects and PD patients. PD samples exhibited unique active pathways compared to controls, including inflammation-related aldosterone and reactive oxygen species metabolism. A comparison of isolated microglia from the HPC or the SN in PD samples showed that biological pathways linked to behavior, regulation of transport and synaptic transmission were among the most differentially regulated ones. Thus, this study was the first to demonstrate microglia transcriptional heterogeneity when comparing different brain regions from control subjects and PD patients.

During the last decade, high-throughput techniques for single-cell RNA sequencing (scRNAseq) have been developed, which have revolutionized our capability to decipher cellular heterogeneity at the transcriptional level. Most of these high-throughput methods rely on the dissociation of the sample into individual cells and can therefore mainly be applied to cultured cells, frozen tissue from young animals or fresh tissue. 

A comprehensive cross-species single-cell microglia transcriptomic analysis in brain tissue confirmed the existence of different microglia transcriptional subpopulations in animals and humans. In addition, the study revealed that human microglia heterogeneity is more complex in humans than any of the tested animal species. Especially microglia pathways suspected to be implicated in neurodegenerative diseases such as the complement system and phagocytosis, as well as PD risk factors are more profoundly expressed in humans compared to rodents as the typical animal model [36].

To circumvent dissociation issues with more mature and solid samples, the RNA sequencing methodology was adapted to single-nuclei (sn). snRNAseq does not rely on intact cells but intact individual cell nuclei. This approach is applicable to frozen human post-mortem brain tissue, the most accessible sample type provided by biobanks (in light of the general unavailability of fresh brain tissue samples from living patients). 

One of the first snRNAseq studies of human cortical and nigral tissue was performed by Agarwal and colleagues [37]. They created a single-cell atlas based on healthy control tissue. Their analysis indicated an enrichment in PD risks variants in neurons. By contrast, they reported no such association in microglia. On the one hand, the lack of PD risk association in microglia may be due to reduced sensitivity of snRNAseq compared to scRNAseq [38] that suggests that snRNAseq might not be ideal to recover microglial states in human tissue. On the other hand, it is possible that exclusively employing control brain samples may mask PD associations in certain cell types. This hypothesis is supported by our own results. Applying snRNAseq analysis to frozen post-mortem midbrain tissue from idiopathic PD patients and controls, we identified a disease-specific upregulation of microglia [39]. In PD patients, activated microglia split into two activation branches; one with GPNMB-high microglia and the other with IL-1β-high microglia. Moreover, employing immunofluorescence and quantitative imaging analyses, we showed that the increase in microglia abundance is restricted to the PD nigra. Additional morphological analyses confirmed that PD patient microglia are more amoeboid supporting cell activation in the SN [39]. Contrary to the findings by Agarwal et al., we observed a significant PD risk variant enrichment in microglia and neurons when focusing on patient midbrain sections. For example, variants in *LRRK2* were highly associated with PD in microglia, which is in accordance with previous research [40].

Overall, very few sc- or snRNAseq datasets from post-mortem brain tissue from PD patients have been published so far, certainly due to the only very recent availability of advanced RNAseq techniques (Table 1). This low number of studies, which typically include only few samples, also does not yet allow for a definitive conclusion about the exact relevance of microglia transcriptional heterogeneity but strongly suggest a functional role of microglia in PD.

Based on the above-mentioned studies, human microglia are considerably more heterogeneous in terms of morphology and transcriptomics when comparing different brain regions than rodents and possibly show disease-specific signatures. These findings highlight the need for further in-depth studies in human tissue to characterize the role of microglia heterogeneity in PD. Beyond human postmortem brain tissue, also the application of single-cell technologies to iPSC-derived patient models may be useful to further elucidate microglia functions and mechanisms in PD (see Section 4.2 for information on this topic).

## 3. (Neuro) Inflammation in PD

With a high degree of neuroplasticity and with low capacity to self-regenerate, the brain is especially sensitive to outer stimuli and injury. In addition, being protected by a blood-brain and blood-cerebrospinal fluid barrier, the brain was considered an “immune-privileged” organ. By contrast, in the last decade, this perception has been challenged and studies have changed our understanding of the immune response in the brain.

Inflammatory processes are considered to be a “double-edged sword”: on the one hand, they help to clear out toxins and unwanted pathogens, while on the other hand, they promote cytotoxicity and neurodegeneration. Several studies suggested mechanisms of immune response and how they can influence neuronal cell death in PD. Already by the end of the 20th century, cytokines and complement proteins, which are components of the innate immune system, were found to be upregulated in brain, cerebrospinal fluid (CSF) and sera of PD patients [41]. Specifically, TNFα, Il-1β and IL6 were discussed as possible PD biomarkers [42,43,44,45]. In addition, Mount and colleagues showed a rise of IFN-γ plasma levels in PD patients compared to healthy controls [46]. Interestingly, the concentration of these markers in peripheral tissues might depend on PD severity and progression. Higher levels of TNFα in serum were characteristic of PD patients with more severe clinical features of impaired cognition, depression, sleep disturbances and fatigue [47,48]. As those studies identified that some of the prodromal symptoms are coupled with an underlying inflammatory response, the question arises whether inflammation really is only a consequence, rather than an active contributor to neurodegeneration.

The adaptive immune response has also been associated with neuropathological features of PD and, initially, the importance of microglial Fcγ receptor was studied. Immunoglobulins from PD patients activated Fcγ receptor positive (Fcγ R+/+) microglia cells in mice, which further promoted dopaminergic neuron cell death, while Fcγ R-/- microglia are not activated and no neuronal loss is detected [49]. There are a number of candidates in PD pathology capable of eliciting immune response and in vitro studies are focusing on components released by dying neurons. Significantly increased levels of autoantibodies were found against neuronal cells, brain lysate, and dsDNA accompanied with significantly higher titers of all three in sera of PD patients compared to healthy individuals [50]. Corroborating this, some studies focused on investigating the amount of mitochondrial DNA (mtDNA) in serum of PD patients. MtDNA, like some other mitochondrial compounds, is considered a damage-associated molecular pattern (DAMP), triggering pro-inflammatory signalling [51,52]. Our own biomarker study revealed higher serum levels of circulating cell-free mtDNA in patients with mutations in *PRKN* or *PINK1* compared to patients with idiopathic PD (IPD) [45]. On another note, studies also showed PD patients having higher autoantibody levels against α-synuclein in blood serum. Those differences highly correlate with inheritance mode of the disease but not with other disease-associated factors, while IPD cases were not significantly different from healthy controls [53]. A different study also showed increased levels of antibodies towards monomeric α-synuclein in serum of PD patients compared to controls, but ELISA responses reduced as PD progressed [54]. Neuromelanin is another neuronal component implicated in the disease. PD patients had elevated levels of autoantibodies against melanin in sera samples. Interestingly, ELISA absorbance response was significantly and negatively correlated with disease duration [55].

With regard to the adaptive immune system, studies investigating postmortem brain samples found reactive CD4+ and CD8+ T cells in postmortem brain samples from PD patients [56,57], suggesting a “porous” BBB and an impact of additional inflammatory mechanisms on the CNS. Furthermore, in the periphery, PD patients have elevated numbers of Th17 cells, which primarily produce IL-17. Interestingly, IL-17 was also found to have a detrimental effect on dopaminergic neuron iPSC-derived autologous cultures [57]. Another study, which explored peripheral immunity in PD, found a reduction in CD4+ T cells in patients. In addition, these cells were observed to preferentially differentiate towards a Th1-reactive state [58]. Recent PD research also showed differentially expressed genes in peripheral blood mononuclear cells (PBMCs), with patients presenting an elevated abundance of CD49d+ Treg cells, which are known to suppress inflammatory processes [59]. When investigating the interaction of microglia and peripheral immune cells in PD, a recent study in rats found an important role of infiltrating T cells. These cells induce a microglial pro-inflammatory response to α-synuclein through upregulation of microglial MHC-II. In turn, this cell-cell communication causes neuronal cell death [60]. Considering the previously mentioned phenomenon of a “leaky” BBB in PD, the above-described phenotypes might be a mere consequence of dysfunctional pathways in brain cells, which release DAMPs into the periphery, causing an ongoing inflammatory loop. It is yet to be investigated how peripheral and CNS immune regulation is achieved in PD. These studies provide insights into the early immune response in PD and highlight novel targets for immunotherapies, which are further discussed in Section 5.

## 4. Disease Modeling: Animal Models, Patient-Derived iPSC Models, and Cell-Based 3D Models and Platforms

**Figure 1:**
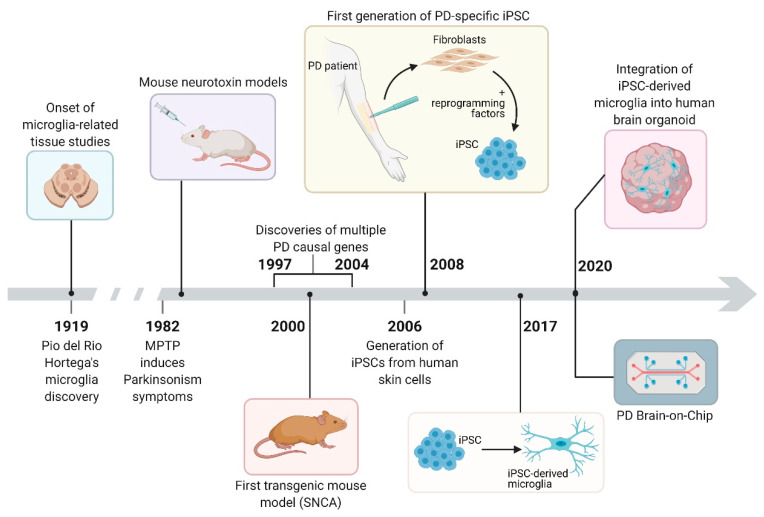
Historical overview of the different model systems used in PD microglia research. Figure created using BioRender.com.

Since the discovery of microglia cells more than 100 years ago, multiple models have been designed to study inflammation in PD. Some of the most widely used models are summarized in Figure 1. In next sections, we will summarize key studies performed in animal and human patient-derived microglial model systems.

### 4.1. Animal Models

PD animal studies may be either of genetic or neurotoxin nature. In vivo animal models are an essential part of pre-clinical trials in drug development. They are highly valuable when investigating the relevance of mutations, tissue pathology, motor symptoms and behavioral patterns in many diseases [61]. In addition, although these models cannot exhibit full neuropathological mechanisms, the progressive nature of PD and sometimes even robust neurodegeneration, they have proven to be invaluable resources of knowledge for researchers. Furthermore, in vitro models include primary microglia isolated from mice or rats, and immortalized murine microglia cell lines like BV-2, which can be greatly expanded and ultimately lead to reduction of animals used in studies. In the next section, we will briefly summarize the inflammatory phenotypes observed in in vivo and in vitro models of PD. 

#### 4.1.1. Neurotoxin Model

Not long after the discovery that mitochondrial respiratory chain inhibition can induce parkinsonism symptoms in healthy individuals, scientists around the world studied the neurotoxic effects of such agents in invertebrate, vertebrate and non-human primates model systems and immortalized cell lines. Among the most commonly used neurotoxins is MPTP, while other studies have also investigated the impact of rotenone or paraquat.

One of the first studies performed in mice with MPTP-induced PD found a significant increase in microglia numbers and altered/activated microglia morphology in the SN early upon treatment, while dopaminergic neurons were depleted days after microglial activation [62]. Furthermore, blocking microglia activation with minocycline (a tetracycline compound with anti-inflammatory properties) after MPTP exposure not only prevented IL-1β formation but also diminished the demise of dopaminergic neurons. Minocycline treatment also reduced MPTP-induced iNOS activity, NADPH-oxidase (both enzymes known for microglia-mediated neurotoxicity), and TNF-α production [63,64]. TNF-α mRNA expression was upregulated in MPTP-treated mice compared to saline-treated animals. This increase preceded the loss of dopaminergic markers such as TH and dopamine, suggesting the involvement of inflammatory pathways prior to neurodegeneration. In addition, upon MPTP treatment, transgenic mice lacking TNF receptors did not exhibit dopamine or dopaminergic neuron loss [65]. Of note, MPTP-treated macaques had increased serum levels of IFN-γ and TNF-α compared to controls, even 5 years after neurotoxin exposure. Monkeys with severe parkinsonism (displaying symptoms of rigidity and a curved posture) showed significant dopaminergic neuronal loss, and activation of microglia and astrocytes [66]. 

Using knockout (KO) mouse models, molecular mechanisms of IFN-γ and TNF-α were further investigated. Upon MPTP treatment, IFN-γ KO mice had a completely rescued phenotype, while TNF-α KO mice had a minor activation of microglia, thus implying a synergistic effect of TNF-α and IFN-γ signaling pathways [66]. IFN-γ was shown to have a significant role in promoting neuronal cell loss after rotenone treatment, but only in the presence of microglia. Treatment of dopaminergic neuron-microglia co-cultures with rotenone revealed that cultures with wildtype but not IFN-γ receptor-deficient microglia had a significant loss of dopaminergic neurons [46]. In rats, rotenone together with lipopolysaccharide (LPS) is initiating neurodegeneration through microglia-mediated NADPH oxidase activation and release of ROS [67]. This NADPH activation was further found to be a direct consequence of HMGB1 (high-mobility group box 1) release from microglia and dying neurons upon ongoing LPS treatment, thereby propagating the detrimental inflammatory loop [68]. Although there have been reports that rotenone cannot directly activate microglia cells [69], additional work confirmed that the activation process is indeed mediated by p38-MAPK signaling [70].

#### 4.1.2. SNCA

**Figure 2:**
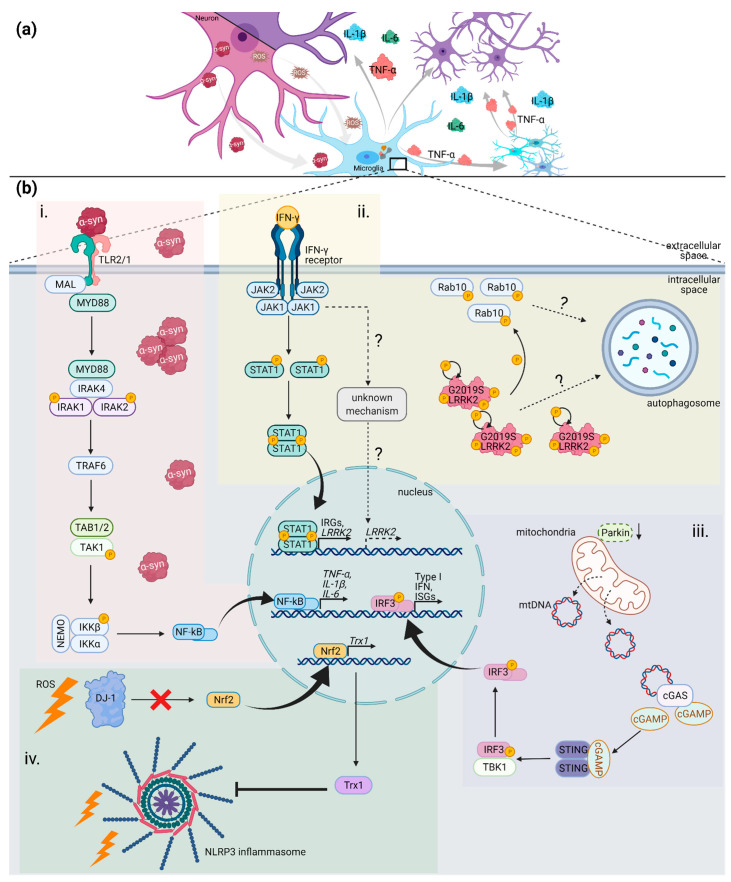
Neuron-microglia cross talk and selected inflammatory pathways involved in PD. (**a**) Inflammatory loop initiated by neuronal demise and propagated by microglial dysfunction. (**b**) Inflammatory pathways associated with α-synuclein (i), LRRK2 (ii), parkin (iii) or DJ-1 (iv) in the context of PD pathology. This figure was created with BioRender.com.

The aggregation of α-synuclein and formation of a-synuclein-containing Lewy bodies and neurites have been established as hallmarks of PD pathology since the first immunohistochemistry analysis of SNpc tissue [13]. Duplication, triplication and point mutations (A30P, A53T, E46K, H50Q, and G51D) in the *SNCA* gene cause an autosomal dominant form of PD [71,72,73,74,75,76]. SNCA mouse models include α-synuclein treatments, overexpression (OE) of human α-synuclein, and transgenic models with inserted human α-synuclein point mutations. Aggregation and neurotoxicity of a-synuclein are subsequent events of oxidative stress, genetic alterations and post-translational modifications like phosphorylation, nitration, ubiquitination and others [77]. Nitrated α-synuclein can activate not only microglia, but also peripheral leukocytes, thus accelerating neurodegeneration [78,79]. Furthermore, when released, a-synuclein can trigger microglia activation and an inflammatory response (Figure 2). In rat midbrain neuron-microglia co-cultures, extracellular α-synuclein had a strong neurotoxic effect, which was mediated by an increase in ROS. Interestingly, neurotoxicity was abolished when microglia were depleted [80]. When priming microglia with α-synuclein injected into the SNpc, mice were more susceptible to environmental insults [81]. This finding suggests a more intricate relationship between the CNS and the peripheral immune response. In addition, both, treatment of primary mouse microglia with α-synuclein and α-synuclein OE in mice, are known to give rise to microglial ROS, TNF-α, IL-1β, COX2, iNOS [82,83]. Furthermore, α-synuclein exposure enhances the gene expression of toll-like receptors (TLRs) as well as of adapters and transcription factors such as MyD88 and NF-κB [84,85]. These results implicate α-synuclein as a damage-associated molecular pattern (DAMP). In agreement with this hypothesis, the activation of TLRs was found to be temporally and regionally induced after α-synuclein treatment, with an early response specifically in the nigrostriatal pathway [83]. Fibrils of α-synuclein but not monomers, can activate the NLR family pyrin domain containing 3 (NLRP3) inflammasome, induce the production and release of IL-1β and cleaved caspase-1, and mediate the release of ASC specks into the extracellular space. Pre-treatment of mice microglia with the small-molecule NLRP3 inhibitor MCC950, ameliorated α-synuclein-mediated inflammation [86,87]. With regard to point mutations in α-synuclein, some studies have shown their greater potential to activate microglia compared to wildtype α-synuclein [88,89,90]. Treatments with mutated α-synuclein triggered diverse phenotypes of microglia activation (based on cellular morphology and pro-inflammatory cytokine expression). While A53T-mutant α-synuclein has shown the strongest effect, wt and E46K had little to no effect on microglia activation. In addition, A53T-mutant α-synuclein is implicated in the NF-κB/AP-1/Nrf2 pathway, which can lead to higher cytokine expression and elevated ROS levels (Figure 2) [88]. Together, these studies suggest a molecular link between α-synuclein aggregation and neuroinflammation in PD. 

#### 4.1.3. LRRK2

Mutations in the *leucine rich repeat kinase 2* (*LRRK2*) gene are the most common cause of PD, having been associated with up to 3% of idiopathic and 5–15% of all familial PD cases [91]. Pathogenic variants include R1441C and N1437H in the Ras-of-complex (ROC) domain, Y1699C in the C-terminal of ROC (COR) domain, and G2019S and I2020T in the kinase domain. Mutations in *LRRK2* are autosomal dominantly inherited. The complex structure of the protein with several active domains may be one reason why the function of LRRK2 is still not fully understood. LRRK2 is predominantly expressed in immune cells (macrophages and monocytes) of the CNS, certain peripheral tissues, and blood, which led to the hypothesis that LRRK2 has its main function in innate immunity [92,93,94]. In this context, it is also important to note that single nucleotide polymorphisms (SNPs) in *LRRK2* have been associated with higher susceptibility to bacterial infections and chronic inflammatory diseases such as Crohn’s disease and leprosy [95,96,97,98]. Unlike other alterations in LRRK2, the most common mutation, G2019S, is not completely penetrant, meaning that not all individuals harboring the single nucleotide change will develop PD [99]. Thus, a “multiple-hit” model was suggested, where a “second-hit” is required for the onset of PD in a G2019S mutation carrier. This model is further corroborated by studies in transgenic LRRK2-mutant mice, which show little to no neurodegeneration [100,101]. Upon stimulation of LRRK2 R1441G transgenic mice with LPS, LRRK2 levels significantly increased [94]. Interestingly, although in primary microglia LRRK2 protein levels were consistently elevated upon LPS stimulation, some studies showed no upregulation at mRNA level, implicating complex post-translational modifications [102]. In addition, LPS-treated transgenic microglia released higher levels of pro-inflammatory TNF-α, IL-1β and IL-6, and lower levels of anti-inflammatory IL-10. The same results were obtained when studying the gene expression of these cytokines, indicating an upstream role of LRRK2 in cytokine modulation. When assessing the neurotoxic effect of microglia-derived molecules, conditioned medium from LPS-stimulated transgenic microglia caused more severe neurodegeneration than medium from LPS-stimulated wildtype microglia [94]. Furthermore, the knockdown of LRRK2 (LRRK2-KD) almost completely abrogated microglial pro-inflammatory phenotypes (with *TNF-α*, *IL-1β* and *IL-6* being significantly reduced) in LPS-treated microglia [103]. These findings in LRRK2-KD microglia were consistent with results obtained after exposure of wildtype microglia to a small-molecule kinase inhibitor, implicating both *LRRK2* expression and kinase activity in the induction of pro-inflammatory mechanisms (Figure 2) [102]. The activation of LRRK2 can be induced through TLR pathways in a MyD88-dependent manner [104]. This finding further illustrates the connection between the innate immune response and LRRK2 function in PD. 

#### 4.1.4. PRKN

Somatic mutations in the *PRKN* gene cause early-onset familial PD, and they are inherited in an autosomal recessive manner [105]. *PRKN* codes for parkin, an E3 ubiquitin ligase, and it is involved in the removal of damaged mitochondria, a process also known as mitophagy [106]. Parkin mutations are rare, but have a strong “mitochondrial phenotype” [107]. The frequency of *PRKN* mutation can reach up to 10–25% in early-onset PD patients, suggesting a strong genetic role in the pathology of PD [108]. Although inflammatory phenotypes in *PRKN*-associated PD have only recently became a research focus, mitochondria have the potential to enable immune responses through several of its components such as ROS, N-formyl peptides, cytochrome c, cardiolipin, and mtDNA, mutually referred to as mito-DAMPs [109]. Deficiencies in both mitophagy and autophagy pathways can lead to the release of these components into the cytosol and the activation of the NLRP3 inflammasome [110,111]. In wt mice, the treatment with rotenone upon an insult with LPS and ATP enabled mtDNA translocation into the cytosol, which, in turn, contributed to the secretion of the pro-inflammatory cytokines IL-1β and IL-18 [111]. First studies in parkin-/- mice could not conclude any differences in neurodegeneration between wt and mutants, prior or even upon acute treatment with LPS. Only upon chronic LPS administration, parkin-/- mice had SNpc dopaminergic neuron degeneration and fine-motor function deficits [112]. The protective role of parkin in immune cells has been reported in multiple studies. Parkin-deficient macrophages had enhanced mtDNA release, mtROS levels, and IL-1β extracellular release upon NLRP3 agonist (LPS and ATP) treatment. Parkin can prevent the accumulation of damaged mitochondria. In Parkin-silenced cells, a buildup of damaged mitochondria was observed upon LPS and ATP insult. The study implicated NF-κB-p62 anti-inflammatory signaling, since parkin recruitment to mitochondria is crucial for p62 tagging within the mitophagy/autophagy process [113]. In addition, parkin is suspected to regulate the transcription of A20, an inhibitor of NF-κB-mediated cytokine expression [114]. Surprisingly, another study found that LPS-treated microglia and macrophages have reduced mRNA levels of *PRKN* and this was prevented by blocking NF-κB signaling [115]. Thus, chronic neuroinflammation can phenocopy parkin depletion, where anti-inflammatory pathways may not be enough to rescue the exacerbated inflammatory phenotype. Furthermore, mtDNA can be detected by the cyclic GMP-AMP synthase (cGAS)—stimulator of interferon genes (STING) pathway. A brief report showed higher mtDNA release in Parkin-/- mice under exhaustive exercise. Moreover, Parkin-/- mice that were crossed with mutator mice (that harbor a defective mtDNA proofreading polymerase) had higher mtDNA release and production of pro-inflammatory cytokines. This increased cytokine expression was abrogated upon STING deletion, implicating mtDNA release and cGAS-STING signalling in the observed phenotypes (Figure 2) [44]. Finally, parkin has the ability to prevent the presentation of mitochondrial antigens on the surface of immune cells by inhibiting mitochondria-derived vesicles (MDVs), linking also autoimmune mechanisms to PD [116]. 

#### 4.1.5. DJ-1

Although *DJ-1* was first described as an oncogene, mutations in this gene are associated with an early-onset PD. The inheritance of *DJ-1* is autosomal recessive and mutations are rare [107]. DJ-1 is expressed in cells with high-energy demand, and it can successfully “buffer” oxidative stress through stabilization of nuclear factor erythroid-derived 2-like 2 (Nrf2) protein [117]. Its role in microglia and inflammatory pathways has not been extensively studied since the focus has primarily been on its expression in dopaminergic neurons, which are particularly energy-demanding cells. Nevertheless, together with its antioxidant activity, some studies concluded DJ-1 has a major anti-inflammatory role in the brain. DJ-1 KO mice had elevated basal levels of IFN-γ in SNpc compared to control mice and this was aggravated after LPS administration [118]. In mouse microglia, DJ-1 can prevent prolonged phosphorylation of signal-transducers and activators of transcription (STAT1) proteins upon IFN-γ treatment by enabling the interaction between Src-homology 2 domain-containing 101 protein tyrosine phosphatase-1 (SHP-1) and STAT1. DJ-1 KO microglia had elevated levels of nuclear p-STAT1, higher expression of *COX-2*, *iNOS*, and *TNF-α* [119]. In addition, DJ-1-deficient microglia had increased mitochondrial activity, which resulted in elevated ROS levels compared to control microglia and those were further exacerbated upon LPS exposure. LPS-treated DJ-1-deficient microglia had higher levels of nitric oxide (NO) and secreted cytokines but lower levels of anti-inflammatory triggering receptor expressed on myeloid cells 2 (TREM2) protein [120]. Although the same group of authors reported higher phagocytosis ability based on zymosan particle uptake, another study showed reduced uptake of soluble α-synuclein in DJ-1 KD microglial cells [121]. α-synuclein could significantly increase the secretion of IL-1β, IL-6, and NO. Also, DJ-1 KD microglia had impaired degradation of p62 and LC3-II upon activation of autophagy. Furthermore, control cells accumulated more α-synuclein upon inhibition of autophagy, while this effect was observed to a lesser extent in DJ-1 KD microglia [121]. Additionally, an extensive study found shared pathways between DJ-1 and NLRP3, which to some extent explains the upregulation of pro-inflammatory cytokines (Figure 2). After MPTP treatment, DJ-1 can suppress NLRP3 activity through upregulation of the antioxidant pathway Nrf2/Trx1, while DJ-1-deficient cells could only be partially rescued by overexpression of Nrf2 [122]. More studies will be needed to further explore the link between DJ-1 regulation of oxidative stress and neuroinflammation.

### 4.2. Human Induced Pluripotent Stem Cell (iPSC) Studies

Advancements in stem cell techniques had a significant impact on the quality of research, especially in neuroscience, since patient material is hard to obtain and animal models in the majority of cases do not fully mirror PD-associated neurodegeneration. Two major classes of iPSC models can be distinguished: gene-modified (in which PD causing mutations are either inserted or corrected) and patient-derived cells. The biggest advantage of iPSC models, which also constitutes their biggest disadvantage, is the genetic background. The original genetic signature of a patient can, on the one hand, help to decipher the potentially multifaceted molecular mechanisms underlying PD in this particular case, while on the other hand, it may cause (functional) heterogeneity between different patient lines. Further information on this topic can be found in a detailed review by Volpato and colleagues [123]. Although, currently, PD studies employing iPSCs are mainly focusing on neuronal differentiation protocols, in the next few paragraphs, we will briefly summarize the available literature exploring iPSC-derived microglia and macrophages in the context of PD.

#### 4.2.1. SNCA

To our knowledge, only one study investigating *SNCA*-mutant iPSC-derived microglia and/or macrophages has been published to date. Differences between control, A53T and *SNCA* triplication macrophages were assessed. *SNCA* triplication cells had higher intracellular levels and an increased release rate of α-synuclein compared to, both, A53T and control cells. The CXCL1, IL-18, and IL-22 cytokine levels in the supernatant of *SNCA* triplication macrophage cultures were significantly upregulated compared to all other investigated cultures. By contrast, there was no difference with regard to the levels of released CCL17, IL-4, IL-6, IL-8, IL-10, and TNF-α between healthy and *SNCA*-mutant macrophages. In addition, in *SNCA* triplication cells, the phagocytic ability was significantly reduced. Further strengthening this result, treating control cells with monomeric α-synuclein had the same impact. Accordingly, high levels of exogenous and endogenous α-synuclein severely disrupt the functionality of iPSC-derived macrophages. The authors found differences with regard to the uptake mechanisms of fibrillar and monomeric α-synuclein [124]. Finally, a very recent study investigated the effect of an α-synuclein antibody on iPSC-derived microglia. Surprisingly, misfolded α-synuclein bound to the antibody triggered inflammatory signalling through the upregulation of the NLRP3 inflammasome [125]. This finding calls into question ongoing clinical trials with antibodies against α-synuclein (see Section 5 for details). 

#### 4.2.2. LRRK2

As described in the previous sections, LRRK2 is highly abundant in immune cells. Thus, iPSC-derived microglia and macrophages are used to explore the impact of LRRK2 mutations on immune signalling in PD. First research revealed that LRRK2 is involved in the later stages of phagosome maturation. Furthermore, the localization of LRRK2 is essential for the recruitment of Rab8 and Rab10 to phagosomes, which is mediated by LRRK2 kinase activity. Moreover, it was confirmed that IFN-γ directly upregulates *LRRK2* gene expression [126]. In another study, LRRK2 G2019S microglia showed increased motility compared to healthy control and LRRK2 KO microglia. This motility reached control levels upon IFN-γ treatment. An assessment of the phagocytic ability showed that G2019S microglia have an increased capacity to engulf fluorescent beads. In addition, LRRK2 G2019S microglia had reduced levels of nuclear NF-kB, upon treatment with both LPS and IFN-γ, suggesting impaired inflammatory signaling mediated by this transcription factor. Moreover, when the supernatant from LPS-treated G2019S microglia was added to control or G2019S iPSC-derived neurons a shortening of the neurite length was observed [127]. Conversely, a study investigating the motility of iPSC-derived monocytes found that G2019S knock-in cells were less motile than wild type cells [128]. In light of opposing findings in microglia, this suggests a cell-type specific impact of the G2019S mutation in LRRK2.

### 4.3. Human Cell-Based 3D Models and Platforms

On the one hand, the complex tissue organization in the brain makes cell-cell communication and interactions difficult to study in in vivo models. On the other hand, in vitro models do not cover the full scope of signalling between all types of brain cells. However, thanks to the development of iPSC-derived co-culture systems, it is now possible to investigate the role of non-neuronal cells in neurodegeneration at least to some degree. Excitingly, 3D cultures, such as brain organoids and brain spheres, have recently emerged as highly valuable tools to model multiple cellular interactions and the pathophysiology of numerous concurrent processes in vivo. Brain organoids have been particularly useful in the research of developmental and neurodegenerative diseases as well as brain cancers [129,130,131,132,133,134,135,136]. By contrast, only a few studies have focused on PD pathogenesis so far [137,138]. Since brain organoids consist of cells from the neuroectodermal region, microglial cells, which originate from the yolk sac, need to be “artificially” added to the 3D culture. While multiple groups have successfully integrated microglia into cortical organoids and brain sphere cultures [139,140,141], some researchers observed spontaneous microglia formation in cortical organoids under differentiation conditions, which do not inhibit non-neuronal lineages [142]. First results suggest that the incorporated microglia support neuronal development and maturation in cortical organoids. Moreover, the microglia appear to become more ramified and matured as the organoid grows [141]. Several research teams, including our close collaborators, are investigating microglial inflammatory signalling in midbrain-specific organoids from PD patients. Though, this work is not without its hurdles. A major challenge of microglia-midbrain organoid cultures is to obtain (and maintain) physiological ratios and distributions of different brain cell types. Significant scientific efforts are currently ongoing to configure cell culture media compositions that will facilitate the simultaneous growth of all cell types, thereby achieving a more physiological microenvironment that prevents the activation of microglia (which is, for instance, commonly observed in the dead core of organoids). Furthermore, cell-based microfluidics systems (also known as Brain-on-Chip technology) have been developed to investigate microglia interactions with astrocytes and neurons under the impact of a continuous fluid flow [143,144,145]. To mimic the BBB composition, some systems are designed to contain a layer of microvascular endothelial cells and pericytes. Exposing a human Brain-Chip (which mirrored the microenvironment of the substantia nigra) to α-synuclein fibrils initiated dopaminergic neuron death, mitochondrial dysfunction and neuroinflammation, while BBB was significantly compromised [145]. Due to their capacity to model the BBB, both organoids and microfluidic systems have emerged as highly promising resources for therapeutic testing in brain diseases [146,147].

## 5. Immunotherapies in Parkinson’s Disease

### 5.1. Anti-Inflammatory Treatments in PD

In a number of animal PD models, the use of nonsteroidal anti-inflammatory drugs (NSAIDs) showed promising results [148]. Exemplary, in an MPTP mouse model, treatment with acetylsalicylic acid, which inhibits the cyclooxygenase isoenzymes COX-1 and COX-2, prevents neuronal loss in the substantia nigra [149]. By contrast, clinical trials exploring the neuroprotective effect of NSAIDs were less conclusive. While several studies reported that NSAIDs may be able to delay or even prevent the onset of PD [148,150], there have also been reports of no impact of NSAID intake on PD risk [148]. A recent meta-analysis that comprised 15 studies on the use of NSAIDs and PD risk found no association. Moreover, a dose-response analysis revealed that the amount and duration of NSAIDs administration did not influence the outcome [151].

In light of these results, alternative approaches that specifically target the transition of microglia from a proinflammatory (M1) to an anti-inflammatory (M2) state have been suggested [148]. Suppression of the M1 state may be achieved by acting at the level of the proinflammatory cytokines TNF-α, IL-1β and IFN-γ [148]. Supporting this hypothesis, the overexpression of dominant negative TNF (DN-TNF) in the nigra of 6-OHDA-treated rats reduced the loss of dopaminergic neurons [152]. An alternative approach may be to modify the endocannabinoid system with the aim to reduce pro-inflammatory microglial toxicity [148]. The expression of the cannabinoid receptor CB2 was found to be increased in glial cells in various conditions affecting the CNS, including AD and PD [153]. Interestingly, while the abundance of CB2 is enhanced in activated PD microglia, an immunolabeling study in human postmortem nigral tissue revealed that CB2 levels are decreased in dopaminergic neurons from PD patients. This observation coincided with diminished numbers of tyrosine hydroxylase-containing neurons in the PD tissue [153]. In line with these findings, administering the CB2 agonist β-caryophyllene to rotenone-challenged rats reduced the release of proinflammatory cytokines which, in turn, prevented dopaminergic neuron demise and the activation of glial cells [154]. 

Also strategies that actively promote a shift of microglia from M1 to M2 have been considered [148]. For instance, the cerebral infusion of a recombinant adeno-associated viral vector expressing human *interleukin-10* (*IL-10*) was tested in an MPTP mouse model. Enhancing the abundance of the anti-inflammatory cytokine led to an increase in striatal TH protein levels [155]. Further of interest in this regard, the multiple sclerosis drug Glatiramer acetate was shown to decrease microglial activation by promoting an M2 state [156]. As in the case of *IL-10* overexpression, treatment of MPTP mice with Glatiramer acetate rescued tyrosine hydroxylase (TH) protein levels in the striatum. Moreover, exposure to the immuno-modulatory agent reversed motor phenotypes in these animals [157]. However, none of the proposed treatments facilitating a shift from M1 to M2 has been tested in humans (for a more detailed review of immuno-modulatory treatment approaches in PD—including a study comparison in tabular form—see Subramaniam and Federoff [148]). Thus, further research in PD patient-derived cellular models will be required to understand the molecular consequences of microglial activation regulators, which will be a prerequisite to eventually use such agents in clinical trials.

### 5.2. Anti-α-Synuclein Immunotherapies

One of the best studied hallmarks of PD are α-synuclein aggregates. As mentioned above, the protein can act as DAMP triggering inflammatory processes. Thus, one research focus in PD is the development of immunotherapeutic approaches that reduce intra- and extracellular α-synuclein levels in the brain. Specifically, antibodies hold the promise of alleviating α-synuclein toxicity and promoting the protein’s degradation [158]. Various epitopes have been tested in preclinical studies with α-synuclein transgenic mice. In two independent studies, administration of antibodies that target the C-terminus (Ab274, 1H7 or 5C1) reduced microgliosis and improved neuronal survival [159,160]. In animals that received ab274, there is even evidence that microglia block the cell-to-cell transfer of α-synuclein by enhancing the clearance of neuron-derived aggregates [160]. With regard to antibodies that are directed against the N-terminus, no improvement of microgliosis phenotypes was observed [158]. Finally, when focusing on antibodies that recognize oligomeric or fibrilar forms of α-synuclein, Syn-O1, Syn-O4 and Syn-F1 effectively reduced protein aggregation and neurodegeneration in transgenic mice [161]. Only animals treated with Syn-O4 displayed decreased microglia levels and showed reduced ramification of these cells in the hippocampus [161]. 

In light of the overall promising results from immunotherapeutic analyses in mice, several antibodies are being tested in phase I clinical trials, which focus primarily on the safety of treatments [162]. By contrast, only two antibodies have so far been moved forward into phase II clinical trials [162], which aim to establish the clinical efficiency of the immunotherapeutics. In the PASADENA study (ClinicalTrials.gov Identifier: NCT03100149), Prasinezumab (PRX002) is currently being tested in patients with early PD. Initial results indicate that the primary outcome of the study was not achieved. At week 52, the MDS-UPDRS total score was not significantly different between the treatment and the placebo group. Nevertheless, some parameters suggest that the antibody delays the progression of motor symptoms in PD patients. However, whether Prasinezumab has a positive impact on microglial phenotypes in these individuals currently remains elusive. In addition, the SPARK study (ClinicalTrials.gov Identifier: NCT03318523), which aims to evaluate the clinical benefits of the α-synuclein antibody BIIB054, is currently ongoing.

## 6. Outlook

The complex and diverse aetiology of PD represents a major obstacle for effective therapeutic interventions. The here described recent findings highlight the importance of neuroinflammatory processes and put microglia as the immanent immune cells of the brain in the focus of novel therapeutic approaches. In particular, this immunological entry point could not only support the identification of common mechanisms underlying the different genetic and idiopathic disease developments but also allow to target disease progression not on the cellular but on the (immune) system level of the brain. While the evidence for the contribution of microglia to disease progression clearly demonstrates the large potential for alternative treatments, further detailed investigations are required to reveal underlying mechanisms and to dissect cellular heterogeneity of microglia in the disease context. Thus, scRNAseq has shown that microglia diversity goes beyond the traditional M1/M2 description and that PD-associated signatures are species- and human-specific but the impact of the microenvironment is still elusive. The recent developments in spatial transcriptomics [163,164] and the application to human iPSC model systems such as organoids [141] and the brain [165] will allow for further insights into the regulation of the complex ecosystem within the brain and how intercellular interactions contribute to PD progression. A major challenge for these studies is the lack of physiologically realistic model systems due to the general limitations of animal models and the not yet resolved issue in human organoid systems, which do not reflect the brain composition of the affected brain regions as described above. Hence, addressing these current limitations is essential to deepen our understanding of the multicellular dimension of PD development and particularly the impact of microglia and their role in neuroimmunology that will not only support the development of new therapeutic strategies in PD but may also provide a more unifying perspective on neurodegeneration given the recent indications for the involvement of microglia in Alzheimer’s disease and multiple sclerosis [165,166].

## Figures and Tables

**Table 1:** Overview of human single cell and single nuclei studies.

Reference	Condition	Samples	Brain Region	Gender	Age (Years)	Tissue State	Method	Analyzed Microglia	Major Outcomes
Mastroeni et al. 2018 [35]	Age-matched control subjects (absence of PD pathology), PD patients	Controls: *n* = 6, PD patients: *n* = 6	SN, HPC (CA1)	All M	controls: 73.6 ± 6 PD patients: 74.6 ± 15.6	Post-mortem frozen unfixed	RT-PCR of immunolabeled (LN3) laser-captured microdissected microglia	Control SN *n* = 3600 microglia cells, control CA1 *n* = 3600 microglia cells, PD SN *n* = 3600 microglia cells, PD CA1 *n* = 3600 microglia cells	Regional heterogeneity (inter- and intra-condition), PD-specific active pathways including inflammation-related aldosterone and reactive oxygen species metabolism
Geirsdottir et al. 2019 [36]	Control subjects (absence of neuropathology)	controls: *n* = 6	Various	3 F, 3 M	9–55	Fresh (surgically removed excess tissue surrounding epileptic focal)	scRNAseq (MARS-seq2.0)	*n* = 1069 microglia cells	Complex human microglia heterogeneity, Neurodegeneration-linked pathways and PD risk factors most profoundly expressed in human microglia
Agarwal et al. 2020 [37]	Age-matched control subjects (no neurological disease)	Controls: *n* = 5	Cortex (middle frontal gyrus), SN (central portion at the level of the third nerve encompassing both ventral and dorsal tiers)	Cortex: 1 F, 3 M; SN: 2 F, 4M	55–70	Post-mortem frozen unfixed	snRNAseq (10X Genomics Chromium Single Cell Kit [v2 chemistry])	Cortex *n* = 500 microglia nuclei, SN *n* = 325 microglia nuclei	PD risk variants in neurons but not in microglia
Smajic et al. 2021 [39]	Age-matched control subjects, idiopathic PD patients	Controls: *n* = 6, PD patients: *n* = 5	Midbrain	Control: 1 F, 5 M; PD: 1 F, 4 M	Controls: 65–95; PD patients: 65–85	Post-mortem frozen unfixed	snRNAseq (10X Genomics Chromium Next GEM Single Cell 3′ Kit v3.1)	*n* = 3903 microglia nuclei	Disease-specific upregulation of microglia, into two branches: GPNMB-high resp. IL-1β-high microglia PD risk variants enrichment in neurons and microglia

## Data Availability

No new data were created or analyzed in this study. Data sharing is not applicable to this article.
